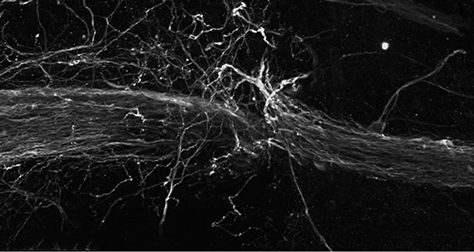# A microRNA mechanism that regulates regeneration after spinal cord injury in amphibians

**Published:** 2014-06

Spinal injuries, which primarily affect young adults, represent a major healthcare burden because there are currently no effective therapies, and treatment largely involves the management of pain and prevention of complications. To mitigate this, researchers are continually working on devising strategies to regenerate nerve cells. Unlike mammals, amphibians are able to regenerate complex body structures after injury. To shed light on the molecular mechanisms that enable regeneration in amphibians, Karen Echeverri and colleagues evaluated changes in microRNA levels after spinal cord injury in the Mexican axolotl salamander and compared them with changes occurring in the rat. They identified a microRNA, miR-125b, that was highly expressed in the salamander compared to the rat. Reduction of miR-125b expression in the salamander was found to inhibit regeneration, via upregulation of miR-125b’s downstream target Sema4D. Intriguingly, increasing the level of miR-125b in the rat resulted in a positive effect on regeneration. The authors conclude that the levels of miR-125b and Sema4D are finely tuned in the salamander to create a regeneration-permissive environment. These findings have implications for the development of regenerative therapies for human spinal cord injuries. **Page 601**

Also in this issue, Catherine McCusker and David Gardiner build on the theme of regenerative medicine by discussing ways that insights gained from studies of salamander limb regeneration could inform the development of cell-based regenerative therapies. Their Review can be read here: **Page 593**

**Figure:**